# Vivisectors and Their Work

**Published:** 1889-04-20

**Authors:** 


### Within the Wards.

### VIVISECTORS AND THEIR WORK.

*(Concluded from page 22.)*

### An Old Hypothesis Experimentally Demonstrated.

It was thus made quite clear that pressure upon the carotid artery in the neck is amply sufficient to check the most serious rupture of a brain blood-vessel which can be imagined. It may still, however, be objected that the carotid cannot continue to be compressed for hours or days in the neck; that some time or other compression must cease, and that then the ruptured artery of cerebral apoplexy will begin to pulsate again, and that the patient will be exactly as he was before. It would be so but for one provision which nature makes against continuous bleeding. That provision is the blood-clot. Human and other mammalian blood when removed from its vessels begins almost immediately to solidify and to form a "clot." The vivisectors found, what they were quite prepared to find, that the blood left at the open mouths of the cut arteries, whilst they were compressing the main trunk in the neck, began to coagulate, and presently formed in the large middle cerebral a firm clot of sufficient size to fill the opening made by their incision. They kept up the compression in the neck until they considered the blood-clot sufficiently firm to resist the powerful pulsation in the vessel which they knew must ensue the moment they released the main trunk. The result was all that could be desired. When the compression was removed the pulse beats returned in full strength, and especially, of course, in the largest vessel, the middle cerebral. But the newly-formed clot remained firm and immovable, and not a drop of blood passed out of the vessel into the brain space. The cure of this worse than apoplectic condition was complete.

### The Application in Practice.

The application is obvious, even to the non-professional mind. Apoplexy, as has been stated, is the rupture of a blood-vessel. When this takes place in the brain, it gives rise to certain symptoms. The symptoms are not produced by the rupture of the vessel, but by the blood which flows out of it. If at the moment of rupture, or within a short time of it—say, an hour or two—any means could be employed which would so check the pulsation in the ruptured vessel as to allow of the formation of a firm blood-clot, the outflow of blood into the brain space would cease, and the apoplexy would for the time be cured. Of course, there would still be dangers to apprehend; but the life could be saved at the most critical juncture, and the other dangers would have to be combatted as they arose. When a man is drowning, the first thing to do is to get him out of the water; the artificial respiration, hot flannels, and other things are seen to afterwards. It is proved, practically beyond dispute, that the carotid arteries in the neck may be compressed in the living subject for a sufficient length of time for a blood-clot to be formed of such a degree of firmness as to resist the outflow of blood into the brain space. That is as much a cure of apoplexy at the critical moment as the pulling of a drowning man out of the water.

### The Nett Result.

How does all this effect the general public? Does it mean that in future not only will apoplexy be a curable evil, but that the laity themselves will be able to deal with it? On the contrary, it means that on the very first appearance of the slightest symptoms of what is called a "fit," except, of course, in the case of known epiliptics, the doctor should be sent for with the speed of lightning. He will be able at once to select the carotid artery of the proper side, and to compress it ordinarily without difficulty. Whilst he is being sent for, the vivisectors suggest that the assistance rendered by the family or attendants should take the form of "first aid," as in the "other simpler forms of accidental hæmorrage so frequently treated with success in public places, and often . . . by persons only trained in ambulance work."

### "In all Things Charity."

Whoever has carefully read through this paper cannot but have seen that whatever may be the painful character of the operations carried out by vivisectors on living animals, the knowledge gained is of the greatest possible service to man, and that the desire for this human and invaluable knowledge lies at the root of all vivisection experiments, and is their chief, perhaps their only, justification. We enter no plea here on behalf of vivisectors; but we do say that all who oppose vivisection should at least try to thoroughly understand the subject they deal with; they should look at it from the point of view of the vivisector himself, and also of mankind at large. The present writer is not a vivisector, and never has been, though he has witnessed many of the gravest experiments made on living animals. He is fully conscious, not only of additions thus made to his knowledge, but of a firm and intelligent hold of physiological verities, which he believes he could have obtained in no other way. In this country the law has so far regulated the practice of vivisection that all unnecessary pain is avoided, and only competent and responsible men are allowed to perform operations and to make experiments. The right attitude of mind for outsiders should be one of unprejudiced candour. We ought all to recognise that vivisectors are men of conscience and honour, men who acknowledge obligations and responsibities, both human and divine; that they must now be left to the free exercise of their own conscientious convictions; and that "to their own master they stand or fall."

